# Variation in near‐surface soil temperature drives plant assemblage differentiation across aspect

**DOI:** 10.1002/ece3.11656

**Published:** 2024-07-24

**Authors:** Elizabeth G. Simpson, Ian Fraser, Hillary Woolf, William D. Pearse

**Affiliations:** ^1^ Department of Biology & Ecology Center Utah State University Logan Utah USA; ^2^ Avian Science Center, Wildlife Biology Program W.A. Franke College of Forestry and Conservation, University of Montana Missoula Montana USA; ^3^ Department of Life Sciences, Imperial College London Berkshire UK

**Keywords:** ecosystem function, environmental heterogeneity, evolutionary ecology, functional composition, functional diversity, plant–climate interactions

## Abstract

Quantifying assemblage variation across environmental gradients provides insight into the ecological and evolutionary mechanisms that differentiate assemblages locally within a larger climate regime. We assessed how vascular plant functional composition and diversity varied across microenvironment to identify ecological differences in assemblages in a mountainous fieldsite in northeastern Utah, USA. Then, we looked at how life‐history strategies and information about phylogenetic differences affect the relationship between functional metrics and environment. We found less functionally dispersed assemblages that were shorter and more resource‐conservative on south‐facing slopes where intra‐annual soil temperature was hotter and more variable. In contrast, we found more functionally dispersed assemblages, that were taller and more resource‐acquisitive on north‐facing slopes where intra‐annual temperature was cooler and less variable. Herbaceous and woody perennials drove these trends. Additionally, including information about phylogenetic differences in a dispersion metric indicated that phylogeny accounts for traits we did not measure. At this fieldsite, soil temperature acts as an environmental filter across aspect. If soil temperature increases and becomes more variable, intra‐annually, the function of north‐ versus south‐facing assemblages may be at risk for contrasting reasons. On south‐facing slopes, assemblages may not have the variance in functional diversity needed to respond to more intense, stressful conditions. Conversely, assemblages on north‐facing slopes may not have the resource‐conservative strategies needed to persist if temperatures become hotter and more variable intra‐annually. Given these results, we advocate for the inclusion of aspect differentiation in studies seeking to understand species and assemblage shifts in response to changing climate conditions.

## Graphical abstract

As climate changes and becomes more variable, north‐ versus south‐facing topography may modulate assemblage response to environmental changes. We used the relationship between vascular plant functional diversity and microenvironment to identify where assemblages are most likely to maintain functionality in a mountainous fieldsite in northeastern Utah, USA. We find that as soil temperature increases and becomes more variable, north facing assemblages may not have the resource‐conservative strategies needed to persist if temperatures become hotter and more variable, while on south‐facing slopes, assemblages may not have the variance in functional diversity needed to respond to more intense, stressful conditions.

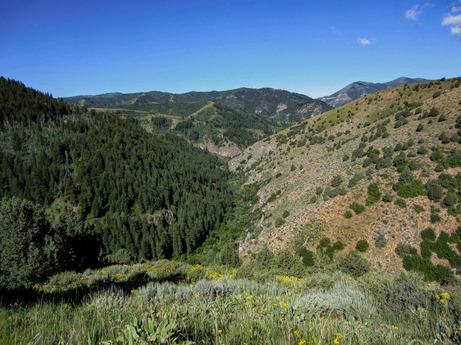

## 1 INTRODUCTION

Identifying the underlying mechanisms that shape assemblage biodiversity provides a foundation for monitoring and management decisions that protect ecosystem functioning (Cardinale et al., 2012; Díaz et al., 2018). As Earth's climate warms and becomes more variable (IPCC, 2018), understanding how biodiversity assembles across environmental gradients may provide critical insight into the types of assemblages that will persist in the future (Lavergne et al., 2010). The biodiversity and structure of an assemblage depend on interacting abiotic and biotic factors and reflect ecological and evolutionary mechanisms that operate at and across spatial and temporal scales (Pavoine & Bonsall, 2011). Quantifying the relationship between functional metrics and environment provides insight into the ways that locally operating ecological mechanisms, like environmental filtering and competitive exclusion, shape assemblage composition and structure (Graham et al., 2014). Assessing how functional and phylogenetic metrics contribute to understanding differences in assemblages across environment provides more nuanced information about how diversity arose and what ecological processes may have shaped assemblage function than species richness or evenness alone (Cadotte et al., 2013; Cavender‐Bares et al., 2006; Pavoine & Bonsall, 2011).

Functional traits and diversity reflect how plants acquire resources and affect the ecosystem around them (Lavorel & Garnier, 2002; Mason & de Bello, 2013; Reich, 2014; Suding et al., 2008). For example, the leaf economic spectrum describes how plants invest resources into their leaves (Díaz et al., 2016; Wright et al., 2004). In resource‐poor, variable environments plants tend to invest resources into leaves that last longer and produce more photosynthate over longer timescales, a conservative strategy. In contrast, plants in resource‐rich environments tend to invest fewer resources into leaves that will not last as long but produce more photosynthate in a shorter time span, an acquisitive strategy. Life‐history strategies classify plants based on their timing of growth and longevity across years in the absence of disturbance (Perez‐Harguindeguy et al., 2016). Within an ecosystem, this classification typically represents species with similar functional strategies (Díaz et al., 2004) and is often shared by closely related species (Díaz & Cabido, 1997, Grime et al., 1997). Life‐history strategies can provide insight into the mechanisms driving species response to change (Adler et al., 2014; Lavorel & Garnier, 2002; Verheyen et al., 2003). If change (e.g., in land use or climate) affects certain life‐history strategies, these life‐history strategies may serve as an effective conservation target that represents desirable attributes within an ecosystem without trying to encompass characteristics of the whole community (Hérault & Honnay, 2007).

The mean and variance in functional trait metrics also provide insight into the factors that shape an assemblage. Individual traits, summarized at the assemblage level as the community weighted mean (CWM) of that trait (Lavorel et al., 2008), represent the central tendency of the plant strategies that have succeeded in an environment. The CWM of traits often shifts as the environment changes, because of phenotypic plasticity and/or species turnover. For example, as summer temperatures warm in the Arctic, assemblages grow taller because of immigration by taller, but still local, species (Bjorkman et al., 2018). However, in the same assemblages, leaf traits only responded to warming at wetter locations, where species invested fewer resources into leaves, potentially allocating those resources to higher growth rates. The variance in all measured functional traits (e.g., functional dispersion; Laliberté & Legendre, 2010) provides insight into the environmental conditions that allowed a greater diversity of functional strategies to succeed. For example, in arid, high alpine meadows in Colorado, assemblages have more variance in plant functional strategies in response to an increase in spatially variable environmental conditions (Stark et al., 2017). However, a global aggregation of local studies found that, broadly, plant assemblages contain less variation in functional traits than expected by chance (Bruelheide et al., 2018). This finding indicates a need to quantify relationships between assemblage function and microenvironment to identify the ways functional traits vary in both composition and diversity across a spectrum of local conditions, from those with more resources and/or less stressful conditions to those with less resources and/or more stressful conditions.

In complement to functional metrics, phylogenetic structure metrics represent the evolutionary relationships between the species in an assemblage and provide insight into the relative importance of the abiotic and biotic mechanisms that shaped an assemblage (Cavender‐Bares et al., 2009; Mayfield & Levine, 2010; Mouquet et al., 2012; Webb et al., 2002). To assess environmental filtering across an environmental gradient, observational studies must include strong support from metrics of assemblage differentiation that include information about evolutionary history. Additionally, assemblages may have more similar phenotypes and/or closely related species than expected by chance in environments that could mechanistically prevent species' establishment (Cadotte & Tucker, 2017). Species' phylogenetic relationships may also represent ecological differences not captured by functional differences (Pavoine & Bonsall, 2011).

**FIGURE 1:**
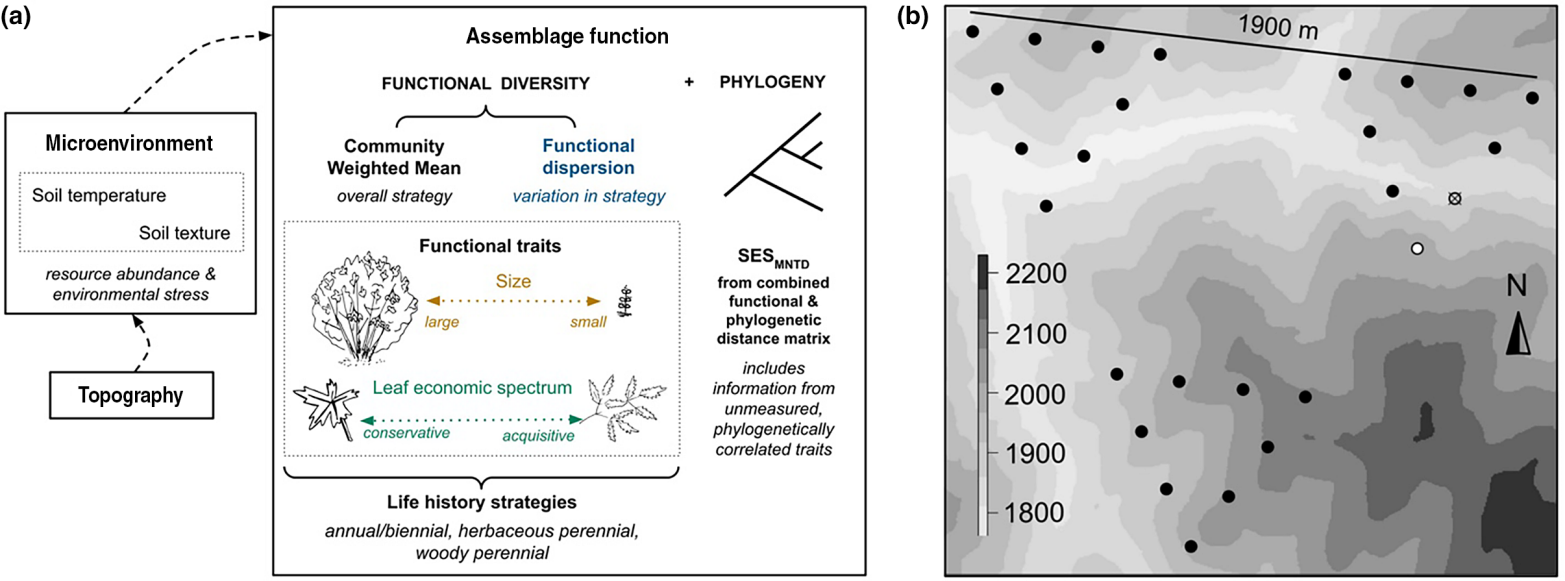
Assessing changes in assemblage function across microenvironment to identify differences in underlying ecological processes in the context of local environmental heterogeneity. (a) Environmental heterogeneity, often driven by differences in topography, differentiates assemblage function and phylogenetic diversity across a range of microenvironments, from those with more resources and/or less stressful conditions to those with less resources and/or more stressful conditions. The CWM of individual functional traits (Lavorel et al., 2008) provides insight into the overall functional strategy individuals in an assemblage utilize, while the functional dispersion in traits (Laliberté & Legendre, 2010) provides insight into the variation in strategies used and/or possible within a given set of environmental conditions. Life‐history strategies—annual/biennial, herbaceous perennial and woody perennial—potentially affect the relationships between these metrics and microenvironments. Additionally, *SES*_*MNTD*_, calculated from the combined functional and phylogenetic distances between the most closely related pairs of species (Cadotte et al., 2013), shows whether phylogeny represents differences between species that were not accounted for in the traits we measured. Color coding of the functional metric text used throughout the figures. (b) We assessed these relationships at twenty‐six 1‐m^2^ plots along the Right Hand Fork of the Logan River in northeast Utah (Simpson & Pearse, 2021). One plot was excluded from the final analysis because the temperature sensor was removed by wildlife disturbance (in white). Background grayscale shows elevation based on a 5‐m digital elevation model in meters (Utah Automated Geographic Reference Center, 2007).

While some environmental changes (e.g., increased moisture) may result in more resources that allow species to persist or colonize an area, other environmental changes (e.g., increased temperature) may stress and/or filter plants. We hypothesize that the mean and variance of plant traits summarized at the assemblage level will reflect differences in resource availability and environmental stress. To test this hypothesis, we assess how the mean (CWM) and variance (FDis) in functional traits shift across microenvironments, defined by near‐surface soil temperature and soil texture, at a fieldsite in northeastern Utah (Figure 1). Life‐history groups encompass similar functional strategies that may succeed or be filtered under different environmental conditions. Therefore, we test whether life‐history groups drive relationships between assemblage function and environment. Finally, metrics of assemblage difference that include information from both phylogenetic and functional sources can provide more information about assemblage differentiation than either metric type alone. We assess this hypothesis by looking at how a metric of assemblage difference that includes both functional and phylogenetic diversity varies across environment. Taken together, our results provide insight into how biodiversity assembled across microenvironments within a broader climate regime at this study location.

## 2 MATERIALS AND METHODS

We assessed how assemblage function changes across microenvironments at 26 1‐m^2^ fractally arranged vegetation plots in a long‐term study site along the Right Hand Fork of the Logan River in Cache National Forest, UT (41°46′12″ N, 111°35′30″ W; Figure 1). At this site, the spatial arrangement of these plots and sampling intensity effectively captured the relationship between phylogenetic diversity and environmental variation across the landscape (Simpson & Pearse, 2021). In that prior work, Simpson and Pearse (2021) found that the spatial scales represented within the sampling design by these 26 plots captured the most variation in the biodiversity relationships assessed, while also optimizing sampling effort. Therefore, we choose to look at how functional diversity varies across microenvironment using the plots at this spatial scale. We assessed the relationship between plant functions and microenvironments in a spatial context, that is, we do not intend to assess the direct temporal response of function to the environment. Additionally, we looked at whether life‐history groups—annual/biennial, herbaceous perennial, or woody perennial—were driving changes in functional diversity across microenvironment. Finally, we assessed whether incorporating information about phylogenetic difference changed the understanding of ecological differences we assessed from functional diversity. Data processing and analyses were performed in R (v. 4.2.1; R Core Team, 2022) and all software packages *in italics* below are R packages unless otherwise noted. All data collected and code to reproduce analyses will be openly released.

### 2.1 Vegetation cover assessment and functional trait collection and processing

We measured the total canopy cover of vascular plant species in each 1‐m^2^ plot during June–July 2018. We included cover from species rooted outside the plot because this best represents the total functionality of the assemblage for abundance‐weighted functional diversity measures. To standardize cover assessment, we used a quadrat divided into four 0.25‐m^2^ quadrants and assessed percent cover with a 10 × 10 grid of 0.025‐m^2^ grid cells. We identified plants using local herbarium resources and field guides and standardized taxa names using The World Flora Online (http://www.worldfloraonline.org/).

We collected functional traits based on their representation of the two main axes of variation in aboveground plant traits at both the species (Díaz et al., 2016) and assemblage (Bruelheide et al., 2018) levels. Plant size (the mean and maximum height) and leaf traits [specific leaf area (SLA) and leaf area (LA)] quantify contrasting functional strategies a plant uses to access light and integrate resources, via competition or facilitation with neighboring individuals (Reich, 2014). We also chose these traits because they both respond to environmental conditions and affect ecosystem functions (Lavorel & Garnier, 2002). To focus on the functional consequences of potentially losing response diversity, we assumed that current variation in functional trait strategies across environment represents a unified functional strategy of response and effect traits, without directly measuring ecosystem function (Elmqvist et al., 2003; Reich, 2014; Suding et al., 2008). All functional trait data were taken in or near the 26 plots during June–July 2018 and June–July 2019 and were collected, processed and analyzed following Perez‐Harguindeguy et al. (2016). We focused on interspecific variation in all traits.

#### 2.1.1 Height traits

Globally, plant height represents the overall strategy of how a species lives (e.g., its lifespan), grows (e.g., time to maturity) and reproduces (e.g., seed mass and the number of seeds it produces; Díaz et al., 2016; Moles et al., 2009). In cold, dry places, like the overall climate at the fieldsite, a wide range of height strategies typically succeed compared to warm, wet environments, where tall species dominate. Because many of the species in the plots are graminoids and forbs, which can be very variable in height, we aimed to measure the height (cm) of up to 25 randomly selected individuals of each species within each 1‐m^2^ plot. If there were less than 10 individuals in a plot, we continued measuring individuals from within 10 m of the plot. We measured from the ground to the top of the main photosynthetic tissue, not including inflorescences, seeds, or fruits if those extended beyond the tallest leaves. Drooping foliage was measured as‐is to assess the general canopy height of the plant. Across all plots, for each species, we calculated one measure of average plant height and one measure of the maximum plant height achieved by that species. Calculating both height measures allowed us to look at an average measure of how height responds to environmental conditions across the site (mean height) compared to the maximum height that species achieved across all the plots.

#### 2.1.2 Leaf traits

We calculated the specific leaf area (SLA), the total fresh leaf area (LA; mm^2^) divided by its oven‐dry mass (g), to quantify the resource acquisition strategy of each species. We aimed to collect at least five leaves from five individuals for each species within 20 m of each plot. We adjusted the number of leaves based on size; from three leaves for large‐leaved species to 20 leaves for small‐leaved species. We collected leaves from each individual randomly, and when possible, chose fully developed sun leaves that were undamaged by herbivory or pathogens. We placed the leaves from each individual in a sealed, plastic bag, to retain their moisture, and kept them flat using cardboard that was tied together for transport back to the lab. The same day, we scanned the leaves with a high‐resolution flatbed scanner. Then, we dried them at 70°C for 72 h and weighed them to determine their oven‐dried leaf mass (g).

We used an automated, threshold‐based pipeline (‘stalkless’ Pearse et al., 2018) to calculate the leaves' surface areas (mm^2^). This workflow relied on thresholding the contrast between dark and light pixels in an image to separate the leaves, or darker areas of the image, from the lighter background. As a baseline, we set the threshold to the mean intensity of each scan plus two times the standard deviation of each scan's intensity. The program identified all regions of the scan greater than the threshold as leaves and calculated LA by counting the pixels in all the regions larger than the mean region size plus two standard deviations as processed LA. We checked all the processed images from the scans and adjusted the threshold to capture the correct shape of each leaf. To focus on interspecific variation in the leaf traits and avoid poor scans, we chose the best sample of leaves from a species, if multiple samples were collected. To choose the best sample, we prioritized fully developed, undamaged leaves, followed by those collected in 2019 when we used a scanner that produced more precise images, and finally, all else equal, focused on samples taken from environments where the species was relatively abundant, and the topography was most consistent with ‘average’ topography at the site.

### 2.2 Quantifying microenvironment

#### 2.2.1 Near‐surface soil temperature

Local seasonal temperature variation directly affects both ecosystem and individual plant functions and relates to other important microclimate conditions, like the consistency of snow cover (Lembrechts et al., 2020). To measure near‐surface soil temperature, we buried a HOBO 8 K Pendant®Temperature/Alarm Data Logger (UA‐001‐08) in a 10‐cm‐deep hole at each of the plots. We anchored the logger into the sides of the hole with metal landscaping pins attached to the logger with zip ties. Then, we covered the logger with soil and rocks, to match the surrounding landscape and protect the sensor from disturbance by wildlife. Each logger was set to record the temperature every 90 min and start logging at midnight the following day using HOBOware software (https://www.onsetcomp.com/hoboware‐free‐download/). We downloaded temperature data during September 2018 and September 2019 to get a full year of temperature data. One sensor was lost in 2018, because of substantial wildlife disturbance (it appeared to be pulled out by a grazer or dug up by a rodent, Figure 1, in white), resulting in temperature data at 25 plots. To summarize the intra‐annual temperature variables, we converted the temperature readings to degrees centigrade using *weathermetrics* (Anderson et al., 2013) and manipulated the date and time to be able to assess the first and last month and day temperature readings recorded at each plot using *lubridate* (Grolemund & Wickham, 2011) and *dplyr* (Wickham et al., 2021). Then, we subset the time frame to a year of temperature data, from September 28, 2017, at 00:00 (Mountain Standard Time, MST) to September 28, 2018, at 00:00 MST, and calculated the annual mean, maximum, minimum, and standard deviation in near‐surface soil temperature at each plot.

#### 2.2.2 Soil texture

We used the hydrometer method [following procedure and calculations in Ashworth et al., 2001, based on Bouyoucos, 1927] to assess soil texture from soil samples collected at the 26 core plots in mid‐summer 2018. At the same position about half a meter from each plot, we removed the organic matter and collected soil from a 10‐cm deep by 8‐cm‐wide hole and transferred it back to the lab to be aired dried for further processing. We physically broke up the soil clumps so that particles would disperse by sieving the soils to two millimeters and further grinding them with a mortar and pestle. We chemically dispersed the soil using a 50 g/L sodium hexametaphosphate solution and finished dispersing the solution by inverting the cylinder several times. We took hydrometer measurements at 40 s and 2 h to determine the amount of sand, silt, and clay in each soil sample. Finally, we used the package *soiltexture* to classify these percentages into soil classes based on the USDA soil texture triangle (Moeys, 2018).

### 2.3 Statistical analysis

We aimed to quantify how biological variation, as measured by functional diversity and phylogeny, relates to environmental heterogeneity, as measured by microenvironment (see the framework in Figure 1). First, we looked at whether differences in topography (aspect, elevation, and slope) predict differences in microenvironment (near‐surface soil temperature and soil texture). Then, we assessed whether topography‐predicted microenvironmental conditions predicted functional diversity—mean and variation. We analyzed whether these relationships differ depending on plant life‐history strategy—annual/biennial, herbaceous perennial, or woody perennial—because these groupings tend to have more similar traits, compared to all plant species. Finally, we incorporated information about ecological differences from both phylogenetic and functional differences to determine if phylogeny quantifies differences between species that were not represented by the traits we measured.

#### 2.3.1 Microenvironment–topography relationships

We looked at how microenvironment varies across topography to quantify how near‐surface soil temperature and soil texture spatially vary across the fieldsite. Since our analysis is based on 25 plots, we aimed to isolate relationships between one microenvironmental variable and one topographic variable to properly estimate coefficients. Soil texture can affect soil temperature (Akter et al., 2015), so we assessed whether each temperature variable correlated with the components of soil texture (percentage of sand, silt, and clay) by calculating the correlation coefficient, Pearson's *r*. Then, we used univariable linear models to assess the relationship between each microenvironmental variable—the mean, standard deviation, maximum, and minimum near‐surface soil temperature and amount of sand, silt, and clay—and the three topographic variables—aspect, elevation, and slope, because drainage patterns can affect soil particle distribution (Brown et al., 2004).

#### 2.3.2 Functional diversity–microenvironment relationships

We quantified the functional strategies in each assemblage using functional dispersion (FDis; Laliberté & Legendre, 2010) and the community weighted mean (CWM, Lavorel et al., 2008) of each of four traits—specific leaf area (SLA), leaf area (LA), mean height, and maximum height. We calculated FDis, the mean distance of all species' traits to the weighted centroid of the assemblage in multivariate trait space, using *FD::dbFD* (Laliberté et al., 2014). We calculated the abundance‐weighted version of this metric to account for how the prevalence of species contributes to assemblage‐level variation in function. First, we generated a species‐by‐species distance matrix from (weighted) functional traits using the Gower (dis)similarity coefficient (Gower, 1971). Because of large differences in the units of the different traits we measured, we standardized each trait to have a mean zero and a unit variance. Then, we performed a principal coordinate analysis (PCoA) on this uncorrected species–species distance matrix to generate PCoA axes that were used as ‘traits'; all four PCoA axes were maintained. To verify that the closely related leaf and height traits were not over‐inflating FDis, we also calculated FDis with just two traits—maximum height and SLA. Again, we used *FD::dbFD* to calculate the abundance‐weighted CWM of the four traits for each assemblage (Lavorel et al., 2008). This provided more detailed information about the functional composition of each assemblage; in the case of the traits we chose, about overall plant size and leaf economic strategies.

To ensure we did not over‐fit our data, we needed to be selective in choosing environmental predictors of functional diversity at our 25 plots. So, we assessed which temperature and texture variable each functional diversity metric temperature correlated most strongly with using Pearson's *r*. We used the most correlated temperature and texture explanatory variable to construct each additive linear model of functional diversity across microenvironment. We modeled the CWM of leaf traits, LA and SLA, and FDis as a function of mean soil temperature and the amount of clay in the soil. We modeled the CWM of height traits, mean and maximum, as a function of the intra‐annual variation [standard deviation (SD)] in soil temperature and the amount of sand in the soil. FDis calculated with two traits, maximum height and SLA, was modeled as a function of mean soil temperature and the amount of sand in the soil. We logged all functional diversity metrics to improve normality and used ANOVA to test whether both, either, or none of the environmental variables best predicted our diversity metrics.

#### 2.3.3 Effect of life‐history strategies on functional diversity–environment relationships

We determined whether each species assessed was a woody perennial, herbaceous perennial, or annual/biennial using a local flora (Shaw et al., 1989), and subset the species in each assemblage into these groups. Then, we calculated all five functional metrics for each of these subsets, as described above. Across the entire site, we calculated the overall FDis and CWM of LA, SLA, and maximum and mean height for each life‐history strategy. Then, at the assemblage level, we used model averaging (using *MuMIn::dredge*; Bartoń, 2022) to statistically test whether each life‐history strategy's diversity and changes across environmental gradients, differed from one another. All predictor variables were z‐transformed to make their resulting coefficients a measure of the relative importance of each explanatory variable (Grueber et al., 2011).

#### 2.3.4 Effect of phylogenetic differences on understanding ecological differences

We assessed whether phylogeny added information about ecological differences using the mean nearest taxon distance (*SES*_*MNTD*_). This metric averages the distance between nearest neighbors for all species in the assemblage and compares that to a randomized, null assemblage drawn from the wider source pool (*n* = 999, Kembel, 2009; Kembel et al., 2010; Pearse et al., 2015; Webb, 2000). We calculated *SES*_*MNTD*_ from the combined functional and phylogenetic distances between the most closely related pairs of species using the phylogenetic weighting parameter a. This ‘traitgram’ approach (Cadotte et al., 2013) means that when *α* = 0, *SES*_*MNTD*_ reflects only functional differences, while when *α* = 1, *SES*_*MNTD*_ is generated from a distance matrix of only phylogenetic differences. Importantly, when a is intermediate between the two, it reflects both phylogeny and traits (when *α* = 0.5 it reflects both equally), and so the relative contributions of both can be assessed.

To calculate phylogenetic distances, we used the phylogenetic tree for vascular land plants from Zanne et al. (2014) which included 73 of the 100 species we identified in our plots (out 31,749 total species in the phylogeny). We added 25 of the missing species using *pez::congeneric.merge* (Pearse et al., 2015) to include 98 of the 100 species we identified in our plots. We set the phylogenetic weighting parameter to calculate abundance‐weighted *SES*_*MNTD*_ 11 times (*α* = 0, 0.1, 0.2, … 0.9, 1), using *pez::.ses.mntd*, to see whether phylogenetic and functional information are revealing related, or complementary, information about our system. Finally, we looked at both how *SES*_*MNTD*_ varied overall, and how the relationship between *SES*_*MNTD*_ and microenvironment changed, as the amount of difference from functional and phylogenetic information varied.

## 3 RESULTS

### 3.1 Microenvironment–topography relationships

**FIGURE 2:**
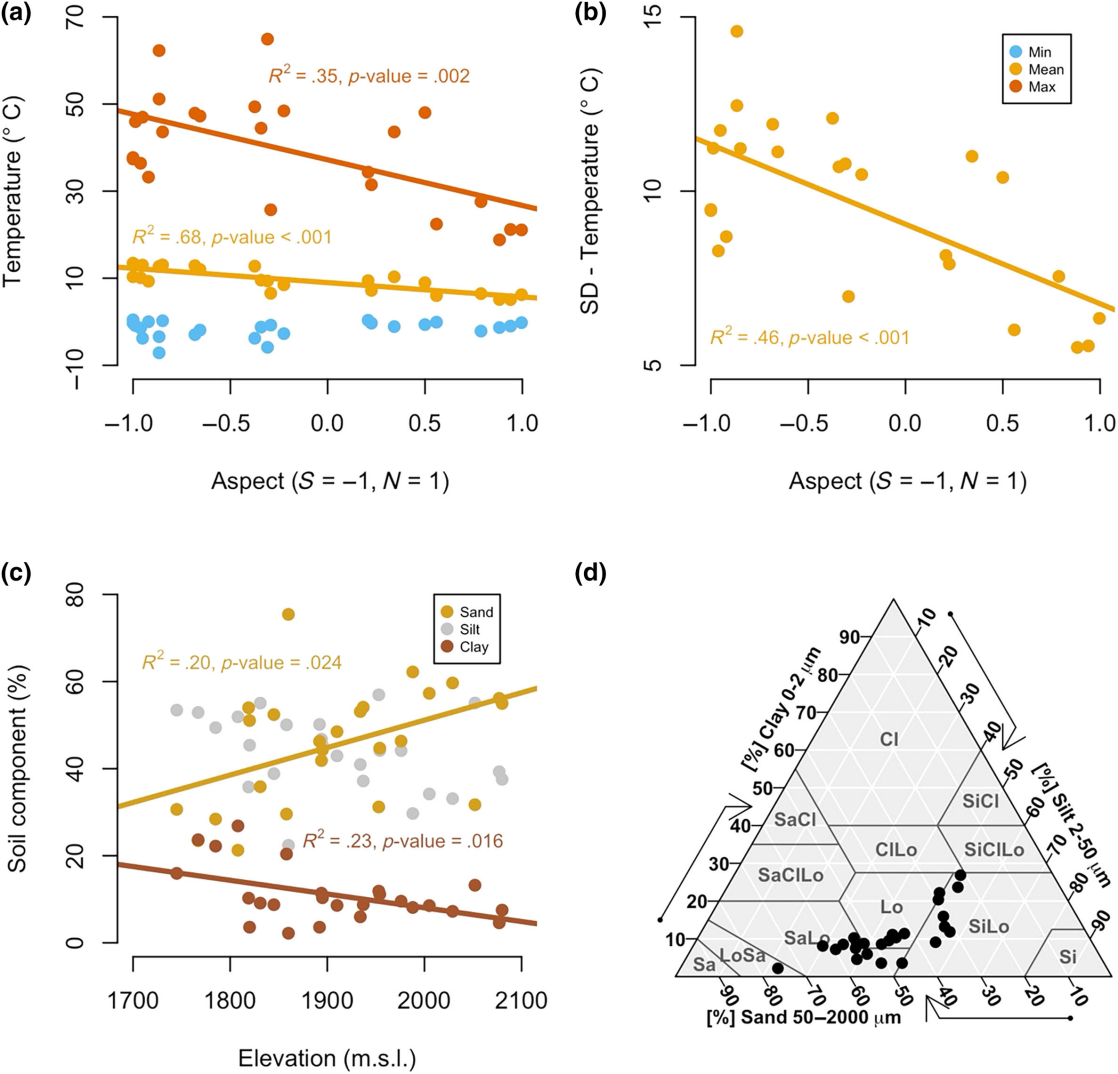
Near‐surface soil temperature and soil texture vary across topography at Right Hand Fork. (a) The mean (yellow, slope = −3.33, *F*_1,23_ = 49.59) and maximum (red, slope = −10.49, *F*_1,23_ = 12.61) near‐surface soil temperatures are significantly higher in more south‐ than north‐facing plots. The minimum temperature does not significantly change across aspect (light blue, average = −1.69°C). (b) The variance in temperature at each plot also significantly decreased from south‐ to north‐facing plots (slope = −2.30, *F*_1,23_ = 19.47). (c) The percent of sand and clay vary inversely across elevation with lower amounts of sand (yellow, slope = 0.063, *F*_1,23_ = 5.85) and higher amounts of clay (brick red, slope = −0.032, *F*_1,23_ = 6.79) at lower elevations. The amount of silt in the soil did not significantly change across aspect (gray, average = 43.70%). (d) All of the soils at Right Hand Fork are loams with lower amounts of clay (0–30%), moderate amounts of silt (20%–60%), and moderate to high amounts of sand (20%–80%).

There were no significant correlations between the two types of microenvironment variables, near‐surface soil temperature, and texture (Appendix S1, Table S1). Soil temperature and soil texture varied across different elements of topography, soil temperature across aspect, and soil texture across elevation (Figure 2, Appendix S1, Table S2). Overall, near‐surface soil temperature variables—the mean, maximum, and standard deviation in temperature—were higher on south‐facing slopes than north‐facing ones. The mean soil temperature ranged from 5.7 to 12.3°C across north‐ to south‐facing slopes. Intra‐annual variation and maximum recorded values of soil temperature followed a similar pattern, with variation in temperature ranging from 6.8 to 11.3°C and maximum temperature ranging from 26.7 to 47.7°C across north‐ to south‐facing slopes. The average minimum temperature at each plot (−1.7 ± 0.39°C) did not vary across aspect.

The soil texture at all plots was loamy, including nine sandy loams, eight silty loams, seven loams, and one loamy sand (Figure 2). The texture of these soils was all low in clay (10%–30%), with moderate amounts of silt (20%–60%) and the highest range in the amount of sand (20%–80%). Both the percentage of sand and clay significantly varied across elevation. Elevation predicted lower amounts of sand (about 35%) and higher amounts of clay (about 15%) at the lowest elevation plots (1745 m.a.s.l.) and higher amounts of sand (about 55%) and lower amounts of clay (about 5%) at the highest elevation plots (2080 m.a.s.l, Figure 2). The amount of silt in the soil did not significantly vary across elevation (average = 43.7%).

### 3.2 Functional diversity–microenvironment relationships

**FIGURE 3:**
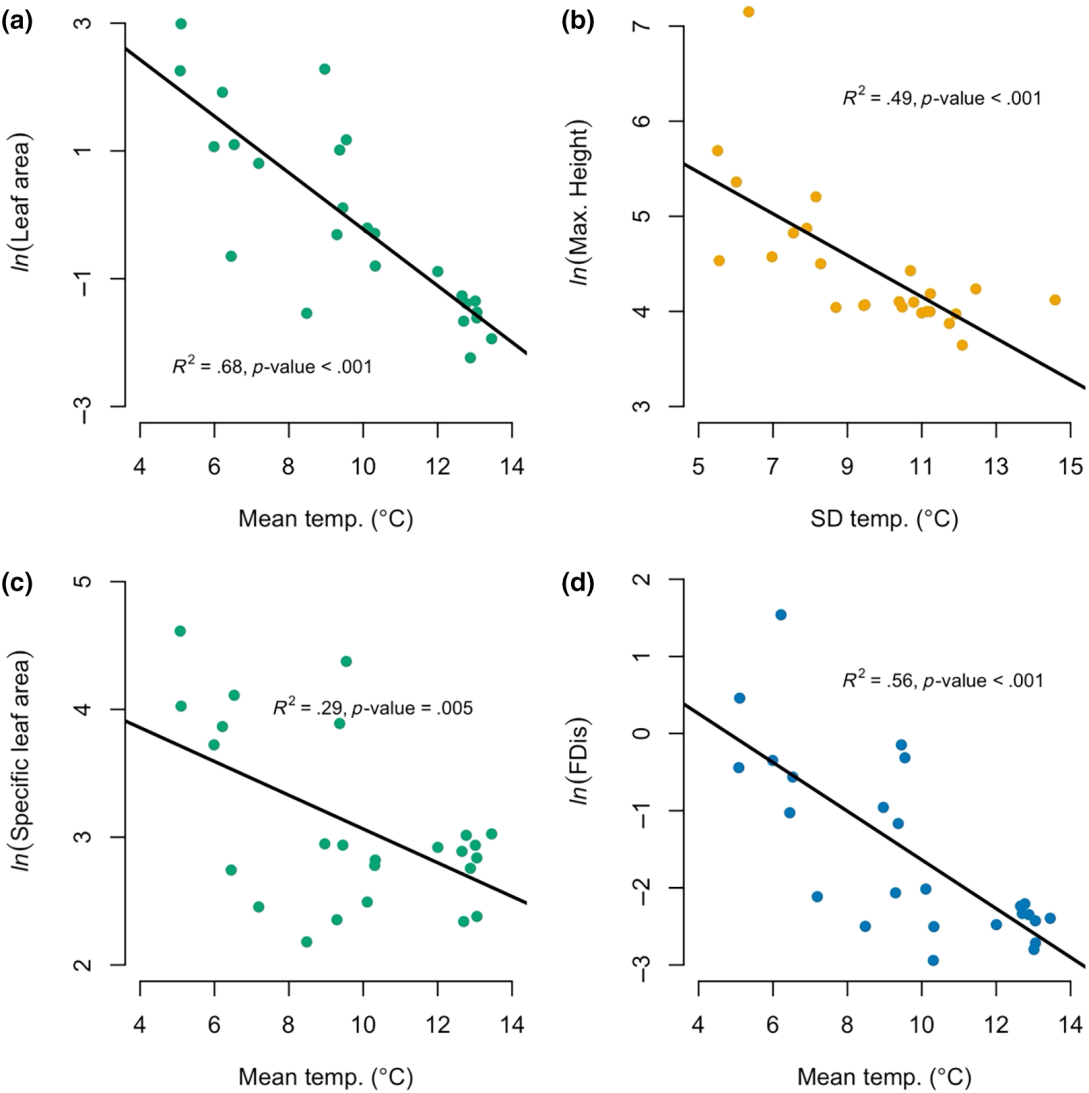
Increases in mean soil temperature predict a decrease in the CWM of leaf traits and functional dispersion, while an increase soil temperature variation predicts a decrease in the CWM of maximum height. (a) Plots with cooler mean soil temperatures support larger leaves [higher logged CWM of LA (mm^2^), slope = −0.442, *F*_1,23_ = 49.97], (c) leaves with more acquisitive leaf economic strategies [higher logged CWM of SLA (mm^2^ g^−1^), slope = −0.132, *F*_1,23_ = 9.426], and (d) assemblages with more variance in functional strategies [higher logged FDis, slope = −0.316, *F*_1,23_ = 29.54]. (b) Plots with less variation in soil temperature support taller assemblages [higher logged CWM of maximum height (cm), slope = −0.218, *F*_1,23_ = 21.85]. Color coding is described in Figure 1.

We obtained four traits for 84 out of the total 100 species identified. We scanned and weighed a total of 7213 leaves and obtained LA from the stalkless pipeline for 6613 of those leaves. Prioritizing the best sample of leaves for each species resulted in 3454 leaves that were used to generate the leaf traits in the analysis presented here. We measured the height of 1831 individuals, all of which were used to calculate the mean and maximum height variables. Microenvironments with the lowest mean soil temperatures, which tended to be on north‐facing slopes, supported assemblages with larger leaves, more acquisitive strategies, and more functional dispersion (Figure 3, Appendix S1, Figure S1). Conversely, plots with less intra‐annual variation in soil temperature, also found on north‐facing slopes, predicted taller assemblages, whether measured as the mean or maximum. When FDis was calculated with two traits, the relationship between functional dispersion and mean temperature followed the same trend found with four traits; assemblages had more variation in traits on cooler north‐facing slopes and less variation in traits on water south‐facing slopes (FDis [4 traits] slope = −0.316, FDis [two traits] slope = −0.321, Appendix S1, Table S3). The relationship between height and the intra‐annual variation in temperature was also similar whether it was calculated as the maximum height a species achieved or the mean height of the species across the site (maximum height slope = −0.218, mean height slope = −0.233, Appendix S1, Table S3).

### 3.3 Effect of life‐history strategies on the relationship between functional diversity and environment

Site‐wide, functional diversity varied across the life‐history strategies—woody or herbaceous perennial, and annual/biennial. Herbaceous perennials made up the largest group of species (54/84) and had the most acquisitive leaves and largest leaf area (Appendix A1, Table S4). Annuals and biennials made up the next largest group of species (17/84) and had the lowest functional dispersion, least acquisitive and smallest leaves, and shortest height. Woody perennials had more than four times the functional dispersion of herbaceous perennials and were the tallest group.

**FIGURE 4:**
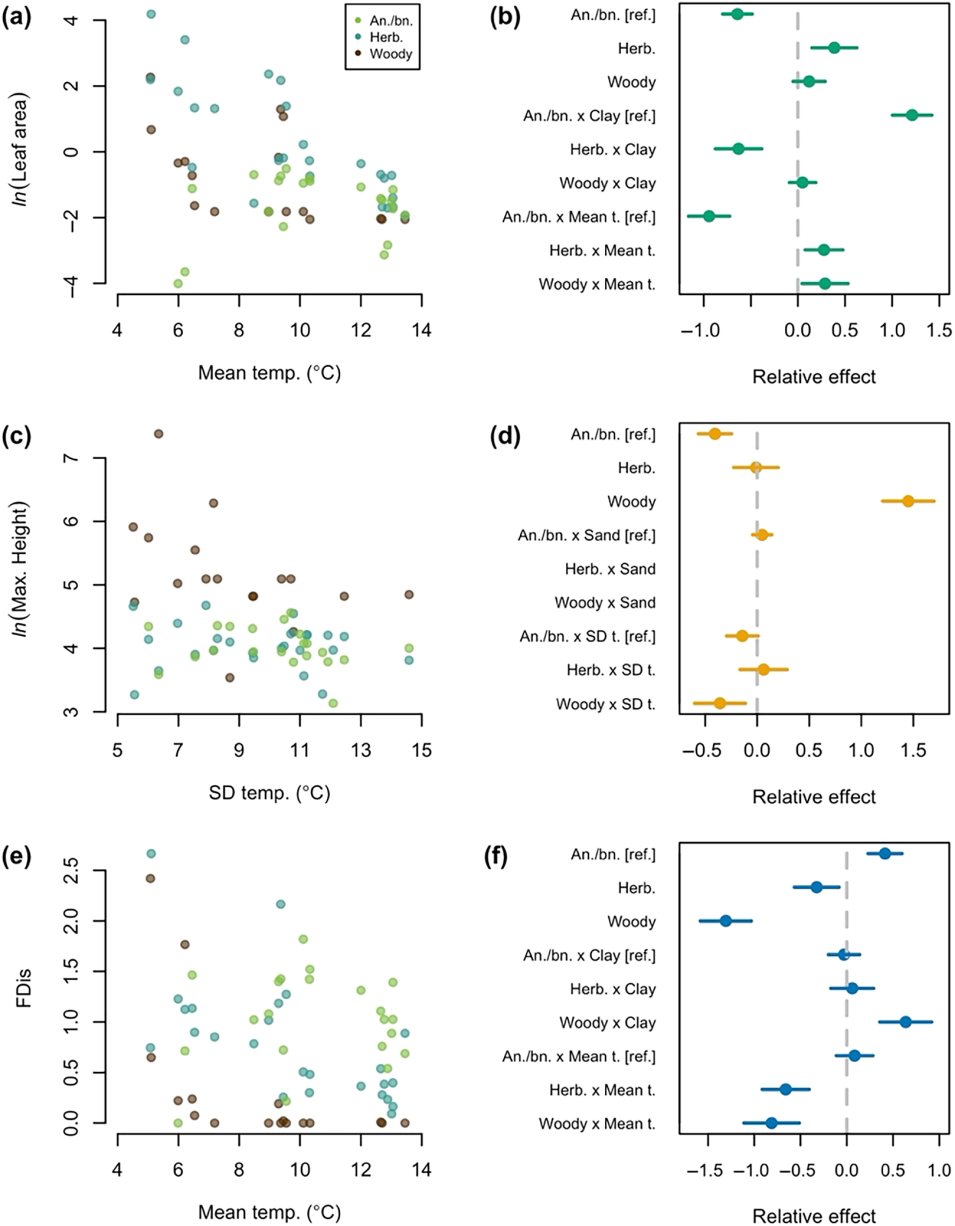
Life‐history strategies affect the relationship between functional diversity and microenvironment. Plots in the left column show how the logged CWM of (a) leaf area, (c) maximum height, and (e) [unlogged] functional dispersion vary across the temperature variable they were most correlated with when subset by life‐history strategy—annuals/biennials (green), herbaceous perennials (blue), and woody perennials (brown). Plots in the right column—(b), (d), and (f)—show the relative effect of each explanatory variable in models that look at how these life‐history strategies affect the relationship between each functional metric (in the left column) and both the soil temperature and texture variable most correlated with that functional metric. Coefficient values are reference contrasts from those labeled as such. Values further from zero indicate that a variable or interaction between variables has a greater effect on a functional metric. For example, when a life‐history strategy interacts with one of the microenvironment variables and has a large relative effect, as woody and herbaceous perennials interact with mean temperature in (b), plants with these life‐history strategies have a larger impact on the relationship between that functional metric and microenvironmental variable.

The life‐history strategy of species affected the relationship between functional diversity and microenvironment. Herbaceous and woody perennials drove the interaction between leaf area and mean temperature and functional dispersion and mean temperature (Figure 4). Both groups had larger leaves and higher dispersion when the mean temperature was lower. Woody perennials had a small effect on the relationship between functional dispersion and the amount of clay in the soil; functional dispersion was higher when the amount of clay in the soil was higher within this group. Herbaceous perennials had the biggest effect on the relationship between specific leaf area and mean soil temperature (Appendix S1, Figure S2); species in this life‐history group had more acquisitive leaves when the mean temperature was lower. The CWM of mean and maximum height was mostly driven by the presence of woody perennials, that is, assemblages containing woody perennials were taller overall (Figure 4, Appendix S1, Figure S2). Additionally, when variation in temperature was lower, woody perennials achieved taller maximum heights. Overall, we did not detect a change in the functional diversity of annuals and biennials across microenvironment.

### 3.4 Effect of phylogenetic differences on understanding ecological differences

**FIGURE 5:**
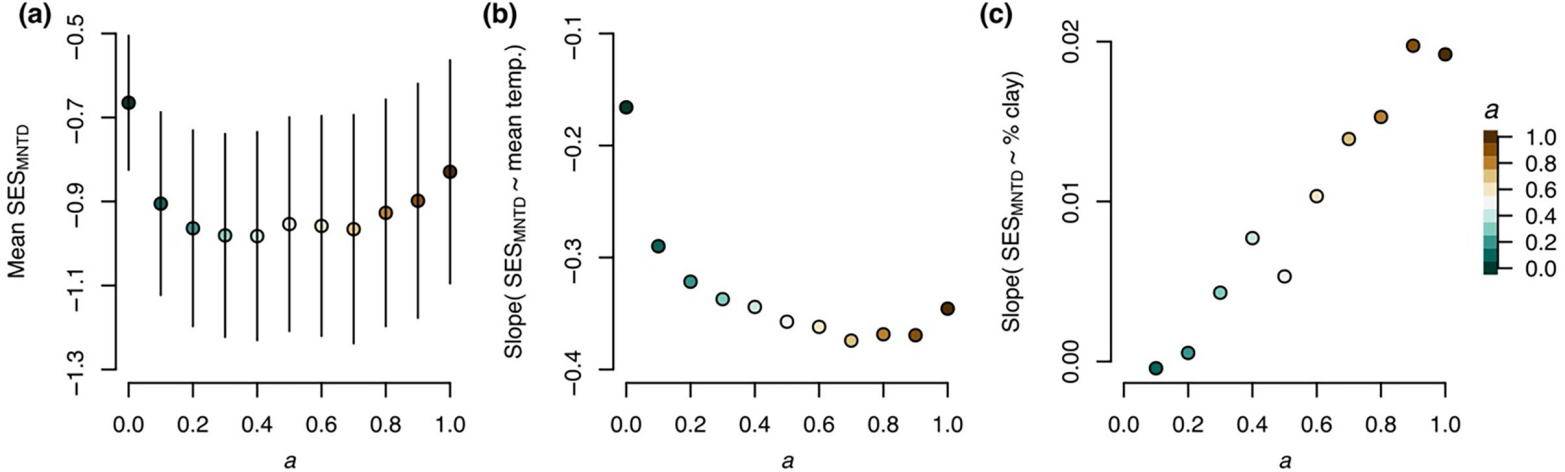
Adding phylogenetic information about ecological differences strengthens the relationship between *SES*_*MNTD*_ and microenvironment. The phylogenetic weighting parameter (a) changes the amount of information about ecological differences used to calculate *SES*_*MNTD*_ from all functional differences (teal, *α* = 0) to all phylogenetic differences (brown, *α* = 1). (a) Overall, including phylogenetic differences does not provide significantly different information about ecological differences than just using functional differences. (b) The relationship between *SES*_*MNTD*_ and mean temperature is stronger when *SES*_*MNTD*_ is calculated from about half functional and half phylogenetic differences or more phylogenetic than functional differences. (c) Similarly, the relationship between *SES*_*MNTD*_ and the amount of clay in the soil is stronger when just calculated from phylogenetic differences.

Broadly, functional and phylogenetic differences between species contributed similar information about the ecological differences between species at Right Hand Fork. Across the whole site, *SES*_*MNTD*_ was highest when calculated only from functional differences (*α* = 0), second highest when only calculated from phylogenetic differences (*α* = 1), and lowest when calculated from about half functional and half phylogenetic difference (*α* = 0.5, Figure 5). However, none of the values of *SES*_*MNTD*_ we calculated across the phylogenetic weighting parameter were significantly different. That said, the relationship between *SES*_*MNTD*_ and environment (both soil temperature and texture) significantly changed as the value of the phylogenetic weighting parameter changed. The relationship between *SES*_*MNTD*_ and mean temperature was least strong when *SES*_*MNTD*_ was calculated only from functional metrics (slope = −0.17) and most strong when calculated from about half functional and half phylogenetic difference or a greater amount of phylogenetic difference (*α* > 0.5, slope = −0.36). Similarly, the relationship between *SES*_*MNTD*_ and the amount of clay in the soil became stronger, albeit subtly, as phylogenetic differences were included (slope = −0.01 to slope = 0.02).

## 4 DISCUSSION

We assessed how functional composition and diversity vary in relation to microenvironment to quantify the factors that potentially contribute to biodiversity assemblage in topographically complex, mountainous terrain in northeastern Utah. Broadly, we found near‐surface soil temperature gradients across aspect and detected shifts in assemblage function across those soil temperature gradients. Herbaceous (and to some degree, woody) perennials had the strongest relationship between assemblage function and soil temperature. Integrating information about functional and phylogenetic differences in a dispersion metric (*SES*_*MNTD*_) indicated that phylogeny represents ecological differences between species that the traits we measured did not represent.

### 4.1 Topography shapes microenvironment

Within our study area, we measured a 6.4°C mean temperature change across north‐ to south‐facing aspects, a shift that maximum recorded temperature and intra‐annual variation in temperature also followed (Figure 2, *R* ^2^ = 69%). Throughout the northern hemisphere south‐facing aspects in mountainous environments receive more solar radiation which increases soil temperature and evapotranspiration (Jackson, 1967). At our fieldsite, we estimated maximum temperatures of 47.7°C on south‐facing slopes. While this temperature may seem extreme, it is common for soil in places with low vegetation and arid climate to have much warmer soil temperature than air temperature (up to 10°C higher, Ashcroft & Gollan, 2013; Lu et al., 2019). At the latitude of our fieldsite (~40° N) south‐facing, 30° slopes receive 1200 W m^−2^ of solar radiation at noon around the summer solstice (Whiteman, 2000) which heats the exposed soil and rock to much higher temperatures than we estimated (62–77°C, Chestovich et al., 2023). However, we did record maximum temperatures at two plots that were within the low end of this range (62.3 and 64.9°C). Notably, these plots still had mean intra‐annual soil temperatures that were similar to the other plots (12.7 and 9.4°C, respectively). All other recorded maximum temperatures were lower than 52°C. Indeed, both of these plots and all plots that recorded high maximum near‐surface soil temperature values (>45°C) were rocky, exposed, and/or had low vegetation cover.

While maximum near‐surface soil temperatures likely contribute to the filtering effect of temperature across aspect, the relationship between mean and intra‐annual variation in temperature across aspect was more predictable. This strong relationship between mean temperature and aspect, especially, shows that small changes in near‐surface soil temperature likely moderate resource availability, like soil moisture, that drive differences in functional strategies. If mean temperatures increase across this site, the intra‐annual variance in temperature and maximum temperatures could become more strongly associated with topography than the mean temperature (Lewis & King, 2017). This shift would indicate that topographic complexity is moderating intra‐annual temperature variation and extremes as part of a new climate regime.

We found variation in near‐surface soil texture across elevation, but not aspect. Soils at higher elevations were coarser with high sand (55%) and low clay content (15%) and overall, soils at Right Hand Fork are characterized by high sand content (>20%, Figure 2). In arid climates, coarser soils allow water to infiltrate to deeper soil layers, thereby decreasing bare soil evaporation (per the inverse‐soil texture effect, Noy‐Meir, 1973; Walter et al., 1973). This leads to greater (overall) water availability in deeper layers that support higher plant productivity and more woody plant growth (Dodd & Lauenroth, 1997; Pennington et al., 2017; Renne et al., 2019; Sala et al., 1997). However, these soils hold less water and nutrients at the surface (Austin et al., 2004). The coarse composition of the soil at Right Hand Fork may support taller plant assemblages than would be present if the soil was finer. To verify this inference, we would need to measure soil depth, soil moisture content, and texture at multiple depths in the soil column because this insight only applies to deeper soils. We could also measure the root traits of herbaceous perennials; if the root traits exhibit more conservative strategies and deeper rooting this would provide support for the inverse‐soil texture effect.

### 4.2 Microenvironment predicts distinct functionally defined assemblages

An increase in the mean and intra‐annual variation of temperature across north‐ to south‐facing aspects supported functionally distinct assemblages (Figure 3). Plots with cooler and less variable temperatures contained taller, more functionally dispersed assemblages that had more acquisitive leaf economic strategies. Conversely, plots with hotter and more variable temperatures supported shorter, less functionally dispersed assemblages that had more conservative leaf economic strategies. This trend indicates that environmental filtering dominates biodiversity assembly on south‐facing slopes, especially for herbaceous and woody perennials. Limited resources (e.g., less water) and environmental stress (e.g., higher temperatures) likely act as this filter, but biotic interactions may also contribute (Cadotte & Tucker, 2017; Cornwell et al., 2006; Kraft et al., 2015; Mayfield & Levine, 2010). Annuals and biennials appear to avoid this filter, potentially by completing their lifecycle in the spring before arid conditions limit their growth and use similar functional strategies across this temperature gradient.

Soil texture did not predict functional differences in the aboveground traits we measured. This aligns with global studies where temperature predicts plant traits more strongly than precipitation (Moles et al., 2014). However, measuring traits more directly related to water acquisition (i.e., root traits) may provide better insight into how microenvironmental variation in water regimes influences observed functional composition and diversity.

Critically, subtle changes in conditions can support assemblages with vastly different community‐weighted trait values (Bruelheide et al., 2018). For example, in a similar arid, alpine environment to our study location, shorter plants with smaller leaves and more resource‐conservative strategies were also found in locations with higher mean and variance in temperature (Stark et al., 2017). However, higher temperatures likely only constrain plant height and result in leaves with more conservative resource acquisition strategies in arid environments where overall water availability is limited. In a study in the Arctic that compared locations with more and less moisture, warmer summer temperatures resulted in taller, more resource‐acquisitive, aboveground plant traits, but only at the wet locations (Bjorkman et al., 2018). Notably, this pattern was mostly driven by species turnover, rather than intraspecific changes in functional traits.

We highlight that environmental heterogeneity, especially aspect, has the potential to provide spatial insurance for assemblage persistence under changing conditions, when microenvironments with less stressful conditions and more abundant resources support species that would otherwise go locally extinct (Greiser et al., 2020; Maclean et al., 2015). Often, species are expected to move up in elevation and poleward in latitude in response to warming temperatures (Rubenstein et al., 2020). However, aspect can potentially buffer the overall effect of warming temperatures, as cooler north‐facing slopes with more functional diversity support site‐wide biodiversity (Albrich et al., 2020; Yang et al., 2020). In response to increasing temperatures, species may also move across aspects and up in elevation, as seen with salamanders and lizards (Feldmeier et al., 2020). Even if cooler, north‐facing slopes do not provide a buffer against increasing temperatures, vegetation often responds differently on north‐ versus south‐facing slopes, which makes this an important topographic characteristic to include in studies of how species and assemblages may shift across environment as climate changes (Ackerly et al., 2020; Elliott & Cowell, 2015).

### 4.3 Phylogenetic difference informs ecological difference

The overall value of *SES*_*MNTD*_, a measure of dispersion, was similar whether we calculated it from solely functional or phylogenetic information (Figure 5). However, the relationship between dispersion and environment was strongest when information from both phylogeny and functional traits was included. This example of how phylogenetic differences can support functional differences [see also de Bello et al., 2017] adds to the ongoing debate about how many axes of functional variation are needed to understand diversity–environment relationships (Laughlin, 2014; Mouillot et al., 2021). While the traits we measured provide information about how assemblages change across environment, the changes we detect are, if anything, conservative.

The strength of these conservative trends provides additional support for our suggestion that future studies looking at how species adapt and move in response to changing environmental conditions should include responses to microenvironmental conditions, like aspect, not just broad climate regimes. This will facilitate a better understanding of subtle species shifts to, or maintenance within, more favorable environments which may help preserve the overall functionality of ecosystems (Fridley et al., 2011). Measuring dispersal traits and monitoring how assemblage function changes over time would further identify the potential for aspect to provide spatial insurance for assemblage function in mountainous landscapes.

## 5 CONCLUSION

Overall, we found evidence for functional differences in assemblages driven by a 6.4°C shift in mean near‐surface soil temperature across aspect, which likely affects water availability on these slopes. Assemblages on the warmer south‐facing slopes had lower function dispersion, more resource‐conservative leaf traits, and shorter stature, consistent with adaptation to a harsher environment than north‐facing slopes. The assemblage‐level traits on north‐facing slopes were consistent with cooler, less harsh environmental conditions. Across the same temperature gradient, adding information about phylogenetic difference to a metric of functional difference strengthened the relationship between ecological difference and environment. These results support the use of functional and phylogenetic metrics to understand differences in assemblages across microenvironments. We suggest that monitoring these relationships over time would provide more information about intra‐annual variability in these relationships. For example, decreases in the FDis of assemblages over time might signal the increasing impact of an environmental filter, like higher temperatures, less precipitation, or land use intensification (Laliberté et al., 2010). Finally, we advocate for more inclusion of aspect differentiation in studies seeking to understand species and assemblage shifts in response to changing climate conditions.

## AUTHOR CONTRIBUTIONS

**Elizabeth G. Simpson:** Conceptualization (equal); data curation (lead); formal analysis (equal); funding acquisition (supporting); investigation (lead); methodology (equal); project administration (supporting); resources (supporting); software (supporting); supervision (lead); validation (equal); visualization (lead); writing – original draft (lead); writing – review and editing (equal). **Ian Fraser:** Investigation (supporting); methodology (supporting); writing – review and editing (supporting). **Hillary Woolf:** Investigation (supporting); methodology (supporting); writing – review and editing (supporting). **William D. Pearse:** Conceptualization (equal); data curation (supporting); formal analysis (equal); funding acquisition (lead); investigation (supporting); methodology (equal); project administration (lead); resources (lead); software (lead); supervision (supporting); validation (equal); visualization (supporting); writing – original draft (supporting); writing – review and editing (equal).

## CONFLICT OF INTEREST STATEMENT

The authors declare that they have no conflicts of interest.

### OPEN RESEARCH BADGES

This article has earned Open Data and Open Materials badges. Data and materials are available at https://zenodo.org/records/10016761.

## Supporting information


Appendix S1.

## Data Availability

We release all data, metadata, and code to reproduce analyses in a public Github repository, https://github.com/elizabethsimps/functional_traits_rhf, which is archived through Zenodo, https://zenodo.org/records/10016761.
